# "The Hospital" Nursing Mirror

**Published:** 1900-11-10

**Authors:** 


"The Hospital" Nursing Mirror.

Being the Nursing Section of "The Hospital."

[Contributions for this Section of "The Hospital" should be addressed to the Editor, "The Hospital" Nursing Mirror, 28 & 29 Southampton Street, Strand, London, W.C.]

### Notes on News from the Nursing World.

### AN OPPORTUNITY FOR THE NEW WAR SECRETARY.

Mr. St. John Brodrick, who takes the place of Lord Lansdowne as Secretary of State for War, has an excellent opportunity of showing that he is abreast with public opinion. Our proposal that a war medal should be given to each of the nurses, British or Colonial, whose services in South Africa have been appreciated by the authorities has been warmly endorsed by several of the daily papers, and, we believe, entirely accords with the feeling of the country. It remains for the Secretary of State to see that it is acted upon, and that the claims of nurses are recognised as fully and freely as those of other persons who have more or less distinguished themselves in the campaign.

### THE WEARING OF UNIFORM ON BOARD SHIP.

An Army nurse puts in a plea in our columns to-day against the compulsory wearing of uniform on board ship. She admits that it may be well for nurses to wear uniform off duty in England, though, as she correctly says, this is still an open question among authorities. But she protests against the application of the rule to nurses on board ship in a tropical climate, under circumstances when a renewal of clean uniform is out of the question, and when the exigencies of the case demand that at the end of the journey the nurse should be prepared to go at a moment's notice anywhere, without reflection on the state of her kit It is, however, the suggestion which she makes at the close of her contribution which we wish to emphasise. She asks that the wearing of uniforms may be made optional on the return voyage from South Africa. This is not a large demand, and we shall be glad if it can be conceded.

### PROFESSOR HUGHES AND THE NURSES.

The death of Professor Hughes is greatly lamented in the nursing world. The nurses who worked under him in the Welsh Hospital were devotedly attached to him, and during the whole of the time he was in South Africa he retained the affection of the entire personnel. Since he arrived at home, stricken with enteric fever, no fewer than four nurses were engaged at Chester Terrace in ministering to him, and it is a melancholy consolation that nothing was left undone, so far as they were concerned, to save his valuable life. He was deeply grateful for the attention paid to him, and when he was conscious was calm and uncomplaining.

### MRS. RICHARD CHAMBERLAIN.

The value of Mrs. Richard Chamberlain's evidence before the South African Hospitals Commission will, no doubt, be duly weighed by Lord Justice Romer and his colleagues. Without in any way anticipating their judgment, we may observe that she herself disposed of any claims to be considered a nurse, notwithstanding that she has been seen in nurse's costume. Just before the enquiry closed, Dr. Church asked her if she had received any hospital training before going to South Africa, and her reply was "No," but "she had been for three years a visitor at the Guards' Hospital, Rochester Row, and had also had experience of hospitals at Birmingham." At the outset of her evidence she stated that she was taken to No. 1 General Hospital at Wynberg by the general commanding the line of communications, and received permission to go into the hospitals three times a week; while, "as a matter of fact, she went every day." She denied that the principal medical officer complained "until about seven months afterwards." If he really was so longsuffering, or so lax in the enforcement of discipline, as Mrs. Richard Chamberlain asserts, we are not surprised that there was a "plague of women" at the front—women who took upon themselves, or endeavoured to take upon themselves, obligations which can only be properly discharged by qualified nurses. Mrs. Richard Chamberlain's ideas of discipline are shown, not merely by the acknowledged defiance of authority already cited, but also in a letter whose authenticity she admitted. Even the long-suffering P.M.O. informed her that he would like himself to distribute the tobacco she had brought for the soldiers. Whereupon she wrote to a friend: "Surgeon-General be blowed. I am going to give the men cigarettes to-morrow, and if Colonel Anthoniss does not like it he can complain to Wilson."

### THE KILBURN SISTERS IN THE FAR EAST.

At present only meagre accounts have reached Kilburn of the work of the Sisters in China. We are informed, however, that the Bishop of Korea applied to the Sister in charge of the Sisters' Mission House in Seoul for temporary help in response to an application he had from Admiral Sir Edward Seymour, and that as the hospital in Seoul was closed at that period she was able to send three nurses to Wei-hai-wei for the time, namely, Miss Unwin, Miss Cameron, and Miss Mills. But they expected, when the last mail came, to return to Korea very shortly.

### QUEEN'S NURSES AT LOUGHBOROUGH.

At the fourth annual meeting of Loughborough District Queen's Nursing Association, the Rev. A. A. R. Gill said that "he had never been in a house in Loughborough where the nurses had been in which they had not met with the highest approval." This is a first-class testimonial, for it would be impossible to praise their efforts more completely. Moreover, it was endorsed by Dr. Phelps, who said that "if the members of the Association really knew what was actually done by the nurses, he was confident they would double and treble their subscriptions at once." As, but for a sale of work which brought in £55, the Association would have been badly in debt, the doubling and trebling process would be most acceptable.

### MADRAS GENERAL HOSPITAL.

According to a correspondent of the *Madras Times* of October 17th, who is indignant at "the attack" on the General Hospital, "the only grievance" that nurses in that institution have at the present time is that "the work is very hard, in fact impossible to do thoroughly"! This strange defender of the Madras General Hospital goes on to say: "When the number of the nurses are compared with that of the patients, this is not to be wondered at—30 nurses for 500 patients, for day and night duty. No night superintendent, no operation theatre nurses, and no relieving nurses for those off duty through sickness or otherwise." We do not see how it would be easy to prefer a more crushing indictment against the institution than this, and the conclusion of the writer that it is merely "for want of thought that the staff has not been increased," does not weaken it. For such cases want of thought is unpardonable, and the statement that the Madras Government "has no more loyal, hardworking, and devoted servants than the nurses"—which we do not question—is but an additional reason why they should not be worked like galley slaves.

### AMSTERDAM NURSES.

An association has been formed in Amsterdam for furthering the interests of male and female trained nurses. The preliminary board of management consists of nurses and medical men; but it is intended that in the future the organisation shall be managed by nurses only, supported by a council of advice. The association has already a journal of it own.

### THE SAME OLD NAMES.

At the eleventh annual meeting of the Dundee Sick Poor Nursing Association one of the speakers who urged its claims to support said that there was much auriferous land in Dundee which had not been mined. "Dundee charity lists," he continued, "were monotonous reading—the same names occurred again and again. If those whose names appeared went for a pleasure sail in a small boat, which they could easily do, they might be drowned, and then the simple upsetting of the boat would bring the charities of Dundee to an end." The same event would, we presume, banish from the homes of the poor "the ray of sunshine," which, according to another speaker, entered with the nurses. There is no doubt, however, that nursing organisations, in common with other beneficent associations, too often owe their continued existence to the few who regularly put their hand into their pocket while the many never contribute a farthing. We can see no objection to the same old names appearing and re-appearing in subscription lists, but there ought to be constant and numerous additions. How considerable the work of the Dundee nurses is may be judged from the fact that in the year ending September 30 no less than 1,915 cases were nursed and 38,162 visits paid.

### NURSES AS FIREMEN.

At a fire which broke out in Goldsmith Row during Monday night at a shop opposite the North-Eastern Hospital for Children, a number of nurses belonging to the hospital came to the rescue some time before the fire engine arrived on the scene. The calm and able manner in which they discharged their duties attests the thoroughness of the drill and instruction they receive in the use of fire-extinguishing appliances. They succeeded, with the help of the three resident doctors, in bringing four powerful hoses to bear upon the building. Owing to the street being exceedingly narrow, the North-Eastern Hospital itself might have been in danger if the fire had been allowed to spread; and there is no doubt that the combined efforts of the nurses and doctors were instrumental in saving the adjoining property from destruction.

### A NEW ARGUMENT AGAINST A NURSING HOME.

The Salford Board of Guardians have decided to act on the recommendation of the Infirmary Committee and build a nursing home on land adjoining Hope Hospital. But an attempt was made to delay the matter for a year or two, and Mr. Wallwork, who objects to the scheme, urged as a reason against proceeding with it the fact that "there were people who went to Blackpool for the benefit of their health, who had smaller rooms placed at their disposal than were in use at the Salford Union Infirmary by the nurses." We presume that the Salford nurses who visit Blackpool for the benefit of their health, or otherwise, are usually guided in their choice of rooms by the amount of money they can afford to expend, and the "analogy" cited by Mr. Wallwork shows the weakness of his case. On the other side the argument was that the proposed improved accommodation was urgently needed. In fact, the chairman of the Infirmary Committee asserted that it was essential if the nurses were to be kept in good health for their work. Nor is the sum involved a very serious one. The entire scheme, of which the nursing home is only a part, will not cost more than £6,000, and will add but a trifle to the rates. Even in spite of the uncontradicted statement of a lady guardian that some of the cubicles for the nurses are no larger than a cab, Mr. Wallwork tried to prevent the adoption of the committee's recommendation. Happily, his amendment was defeated on a divison, but his attitude, and that of four other members of the Salford Board, shows how difficult it is to get the average guardian to realise the responsibilities which they owe to the nurses in their employ.

### THE MEMORIAL TO THE FOUNDER OF THE DARLINGTON NURSES' HOME.

On Friday last the public tributes to the memory of the late Mr. Arthur Pease, M.P., were completed. They have been threefold in character, consisting of the presentation to the town of a life-like portrait of the late member; the freeing of the Queen's Nurses' Home from debt; and the insertion of a tablet in the walls of the Home. The tablet occupies a position on the north side of the main entrance of the Home, and has been carved out of an exceptionally fine block of Hill of Fair red Scotch granite. Sir David Dale, Bart., presided over a large attendance at the unveiling ceremony, which was performed by Sir Joseph W. Pease, Bart., M.P., brother of the deceased. Sir Joseph, in his address, bore testimony to the energy of the Queen's Nursing Sisters, whom, he said, worked not only for the benefit of the working classes, but of all classes in that neighbourhood. The Home started work on April 1st, 1892, at the suggestion of the late Mr. A. Pease, with one trained nurse from the Queen Victoria Jubilee Institute for Nurses, with which it is affiliated. The staff now consists of four, the annual cost of the Home being about £400. The founder contributed £100 per annum towards its funds. The Home cost £1,475, partly raised by subscriptions, and at the death of Mr. Arthur Pease there was a balance due of £609 19s. 3d., which has since been liquidated.

### A NOVEL ENTERTAINMENT.

A novelty in the way of entertainments is promised at Hammersmith Town Hall on Wednesday evening, November 28th, when an entertainment will be given in aid of the Hammersmith and Fulham District Nursing Association. After a series of sacred tableaux with music, scenes from the life of a district nurse will be portrayed

The following is the syllabus:—(1) The daily start; (2) The nurse's welcome; (3) In the Board Schools; (4) The gates of death; (5) Given back; (6) After the day's work.

### NATIVE JAPANESE NURSES.

One of the features of the Japanese hospital ship which was fitted up in connection with the Chinese crisis is the native nurse. There are, it seems, a dozen "little maids from school" who have entered upon the serious business of nursing, and they take it seriously. They are all said to have been to high-class colleges, learnt English and medicine, and practical nursing up to date. Their dress, in its simplicity, is in striking contrast to that ordinarily worn by Japanese ladies; and their rules for behaviour are much more Draconian than those of their English sisters. It is stated that if a man walks past them on the deck of the ship they all rise at once and bow respectfully; and that if their matron has occasion to speak to an officer in the ship, she begins with a most profonnd bow, which he acknowledges with a faint nod.

### NURSE DISPENSERS.

In our issue of October 6th, commenting upon the decision of the directors of Arbroath Infirmary to appoint a nurse qualified to dispense, we pointed out that a nurse who, in addition to her ordinary training, has been at the expense of learning how to dispense is entitled to a higher rate of payment. It is satisfactory to learn that this view has commended itself to the authorities of the Shanghai Nursing Home. They are offering a nurse who can dispense £25 a year in excess of the annual salary of £75. If it is to become a practice to increase by a fourth the salaries of nurse-dispensers, the example will certainly soon multiply.

### A WORKING MAN'S PRESENT TO A DISTRICT NURSE.

The most interesting episode at the annual meeting of the Helston District Nursing Association was the reading of a letter from a working man, who wrote to Nurse Smith offering for her acceptance a bandage-rolling machine in grateful appreciation of her kind and skilful nursing of his son when he was laid up with a severely injured head. In addition to making 2,3C6 visits last year, Miss Smith gave instruction in nursing to classes of girls belonging to Helston and Porthleven schools. There is, happily, a balance in hand.

### NURSE DOWLING'S ACTION.

Last June Miss Agnes Dowling obtained a verdict for £100 in a libel action against Dr. Louis Dods. The defendant appealed, and the case was heard on Tuesday. After hearing counsel on both sides, the Master of the Rolls gave judgment for the appellant, in the course of which he said that he could not see the slightest evidence that the defendant was acting with malice towards the plaintiff when he sat down and wrote the letter to the relieving officer which formed the basis of the charge. He therefore thought that Mr. Justice Darling, before whom the case was tried, would have been well advised if he had ruled not only that the occasion was privileged, but also that there was no evidence of express malice to go to the jury.

### A SILLY JOKE.

At a meeting of the Hayfield Board of Guardians last week the question of the scarcity of workhouse nurses was discussed. It was reported that for about two months past the Board had spent from £1 to £1 10s. each week in advertising for a nurse, but had been unable to obtain the services of one. Two applications had latterly been received, but these were not from certificated nurses, and it was decided to again advertise. An application for the appointment had also been made by Mr. John Lowe, of Birch Vale, an ex-member of the New Mills District Council. It was ordered that the letter "lie on the table." The proper place for it was the waste-paper basket.

### OUR CLOTHING DISTRIBUTION.

We venture again to remind our readers that articles for our Clothing Distribution at Christmas should reach us not later than December 17, and should be addressed to the Editor. Last year some of our kind friends forwarded their parcels after the date named, but it is a great advantage to be able to allocate the whole of the gifts intended for the patients before Christmas. It is because we know how quickly the weeks slip by that we call attention to the fact that there is now little more than a month for the workers to turn to account.

### THE NEEDLEWORK GUILD.

The value of the work done by the Needlework Guild has been warmly acknowledged by nurses and their patients in South Africa. It is in fact impossible to exaggerate the benefits derived by our soldiers from the contributions of clothing and comforts sent out under the auspices of Lady Bradford's admirable association. At the annual meeting the other day, it was stated that altogether about 13,000 garments were shipped by the Guild to South Africa, and many letters of gratitude have been received from those among whom they were distributed. It is satisfactory to learn that the special efforts on behalf of the troops had no effect in diminishing the amount of good work in the Guild's regular sphere of activity, and that, in fact, they collected in the year more articles than ever for distribution at home.

### THE QUEEN'S NURSE AT DUNMOW.

There is one unpleasing feature in connection with the Dunmow and District Nursing Association. Miss Amy Hughes, of the Nurses' Co-operative Association, who gave an address at the annual meeting presided over by the Countess of Warwick, remarked that she did not see in the list of subscribers the Dunmow Board of Guardians. As Miss Hughes proceeded to add, in many places the Guardians are contributing to the Nursing Associations, and the Local Government Board have given their assent because the nurse often attends people who would otherwise have to go to the workhouse. It may be hoped that the Dunmow Board will promptly take the hint, and thus render the best assistance they can to Lady Warwick and Mrs. King in their generous efforts to provide for the needs of the suffering poor. It is largely through their substantial support that Dunmow is able to possess a Queen's nurse, though we note with satisfaction that last year the patients' contributions amounted to £6 11s. 6d.

### SHORT ITEM.

Miss Boileau, Society of the Industrial Law Committee, wishes it to be known that the Committee are very anxious to arrange for addresses to further audiences of nurses. If any heads of institutions or associations who were present at the meeting last month were sufficiently interested to feel that it would be well if the nurses working under them could listen to a similar address, they should be most glad to supply a speaker. They particularly wish to reach district nurses.

### Lectures on Nursing for Probationers.

By E. MacDowel Cosgrave, M.D., Lecturer to the Dublin Metropolitan Technical School for Nurses.

### No. XVIII.—FOOD.

A certain amount of food is required to keep the body in health; for a person doing work about $\frac{1}{100}$ of the body weight has to be taken in daily.

Food has three uses:—To replace worn-out tissues, to supply force, to supply heat. In growing people food is also wanted to supply material for the extra tissues.

Some of the food taken is built up into fat, which can be drawn upon as fuel as the body requires it; more is temporarily stored up in the liver.

There are five classes of food, all of which are necessary for life:—

I. Proteids.
II. Carbo-hydrates.
III. Fats.
IV. Salts.
V. Water.

These are represented in milk (which is a perfect, or complete food) in the following proportions:—

| | | Per cent. |
|---|---|---|
| I. | Caseine | $4\frac{1}{2}$ |
| II. | Sugar | 4 |
| III. | Fat | 5 |
| IV. | Salts | $\frac{1}{2}$ |
| V. | Water | 86 |

I. Proteids.—These contain nitrogen (in addition to carbon, hydrogen and oxygen), so they are capable of building up muscular tissue; they are often called "flesh-forming foods," but this is not quite correct, as they are also used in the body to form heat.

As examples of proteids may be given—lean meat, fowl, game, fish, eggs, milk (caseine), cheese.

Proteids exist in vegetables—oatmeal, peas, beans, and lentils are especially rich. The gluten of grains is a proteid.

II. Carbo-hydrates.—These are starches and sugar; they are made up of water and carbon and have no nitrogen, so cannot by themselves build up the flesh. Carbo-hydrates and fats are often called "heat-givers."

Starch is the most important of the two groups of carbohydrates; it is found in flour, rice, potatoes, arrowroot, sago, tapioca, &c. In the process of digestion starch is converted into sugar.

Starch requires prolonged cooking in order to make it digestible—in fact, the longer it is cooked the more digestible it becomes.

In this way toast can often be taken when bread would disagree; biscuits, which as the name implies, are "twice cooked," are therefore digestible; as are rusks, which undergo three processes of cooking, being first baked as a sponge, then cut into slices and toasted, and lastly slowly dried.

Bread is generally made by mixing yeast in the dough and placing in a warm place; the yeast gives off carbonic acid gas and so breaks up the dough into a spongy mass. It is these openings that allow the heat to get all through the loaf, and so enables the inner part to be cooked.

Baking powders contain tartaric acid and bicarbonate of soda; these, when moist, give off carbonic acid gas. Soda and sour milk have the same effect. There is a popular belief that bread raised by yeast leads to indigestion, but there is no ground for it, as the heat of baking kills the yeast cells.

Wholemeal bread contains more of the flesh-forming elements than bread made with fine white flour; but it also contains more bran, and it is doubtful whether on the whole more nourishment is derived from it.

Breads of the "Hovis" class are free from bran, and yet contain more gluten, and so are of more flesh-forming value; the gluten gives them an underdone taste, which is improved by toasting.

Starches are unsuitable for infants, as for the first six or eight months of life saliva has no digestive power. Cases of starch dyspepsia are also met with; here malted and other predigested foods, or starchy food sweetened with malt extract, may agree. The predigesting preparation or malt extract must not be added until the food is cool enough to take, as a higher temperature will destroy their efficacy. In enteric fever starch should not be given; indeed, in most cases of fever, starchy foods do not agree.

The chief varieties of sugar are cane sugar, milk sugar and grape sugar. Sugar-candy and crystalline sugar are the purest forms of cane sugar.

III. Fats.—Fat is a necessary food, for whilst it can be built up from other kinds of food, this can only be done by the expenditure of much force; so in weakened conditions of the system it becomes practically impossible. Unfortunately in these very conditions there is often great distaste to fat, and it is hard to introduce it into the diet. This is a grave difficulty, as often one of the first signs of serious delicacy is the disappearance of fat.

One of the commonest objections made to taking fat is that it sickens; this is generally caused by the fat melting out and running to oil in the stomach.

Beef fat is more digestible than mutton fat, as it is distributed through the lean fibres and gets better mixed with the other contents of the stomach, so its particles are not so likely to run together.

Mutton fat lies in masses apart from the lean fibres, and frequently disagrees. If cold mutton be eaten with bread or potatoes, the fat gets mixed with the starch and does not so readily run to oil.

Bacon fat is specially useful; it agrees with weak digestion and will be taken when other fat is rejected. Bread fried in bacon fat is capital food for delicate children.

Butter is also a useful form of fat. Sometimes children do not care for it; if, however, two thin slices of bread be buttered, and placed, buttered sides together, it will generally be taken.

Toffee is a capital means of getting children to take butter, and the sugar it contains is good food.

Milk is specially needful for those who will not take butter.

Yolk of eggs contains nearly one-third of its bulk of fat. It is, however, so concentrated that it does not always agree; this can be remedied by beating it up with some fluid.

Cod liver oil is much used in cases of delicacy; it is an easily assimilated fat, and is often taken by those to whom other forms of fat are repugnant. It has, however, been noticed that though cod liver oil puts on fat, the fat so put on rapidly disappears when the cod liver oil is stopped.

If cod liver oil cannot be taken plain it may be given as an emulsion, that is, broken up into globules, as the fat is in milk. The best emulsion is one made with malt extract, as this adds to its food value.

IV. Salts.—Salts of Lime are required for the bones; iron is required for the red corpuscles of the blood, and for the colouring matter of the muscles; phosphorus is required for the brain and nerves; potassium and sodium are required for the tissues and fluids of the body.

(*To be continued.*)

### Nursing at the Palace of Justice Hospital, Pretoria.

By an Army Sister.

"Four weeks after leaving England," writes a correspondent, "I found myself at the Palace of Justice Hospital, Pretoria, now holding almost 500 beds. These include 150 of the Irish Hospital, whose staff was the first to take possession of this fine building, ten days from the time of Lord Roberts' occupation of the town. The installation of electric light and water supply were added when the Irish Hospital undertook to render it fit for the sick, and though not designed for its present purpose its splendid space has been wonderfully utilised for the nursing of patients—the ventilation of the ground floor being, perhaps, its greatest drawback. The huge hall—called Victoria—accommodates seventy-two beds, divided into three wards. Some idea of the general effect may be gathered when it is stated that the hall contains 60 pillars supporting a gallery which runs all round the building and across the main hall in two places. Opening from this gallery and from the sides of the ground floor are many other wards of various sizes named after distinguished men—military and civil—Roberts, Kitchener, Iveagh, Maxwell, &c. Many of the beds have spring frames with straw mattresses, the latter a clean and ready, if not a very comfortable, contrivance. The Irish Hospital beds have scarlet blankets for quilts; always an effective colour, where there is plenty of space. The operating theatre, pharmacy, and other offices are on the ground floor, as also accommodation for sick officers. On the third floor of the building are yet more wards and the sisters' and doctors' quarters.

### The Accommodation for the Nurses.

There is not much room for the staff, and three, four, or even six sisters may have to sleep in one room. Of course we have no wardrobes, and only latterly, about the time of the advent of the Commission, chests of drawers. The bed coverings, too, are very primitive and funny, the place of blankets and quilts being taken by table covers of all textures, curtains, pieces of woollen brocade, curious Oriental hangings and rugs, and pillows proper are supplemented by cushions of many and various hues and shapes. However, the view from the window compensates for other discrepancies.

### The Meals.

The entire staff mess together—a very pleasant and sociable plan. The mess room seats 42, and about 22 of these are nursing sisters, including 8 Canadians, 1 unattached, and the Matron and 1 Irish sister, all the rest being Army Reserve Nurses. Breakfast is at 8, lunch at 1, tea at 4, and dinner at 7 p.m. It will, no doubt, be surprising to hear that the menu for all meals, even in Pretoria at this stage of the war, is a very limited one. Porridge, with a kind of scrambled egg, bread and jam, with an occasional change to uneatable bacon, make up breakfast; lunch always consists of stewed meat, with a second course of bread and jam with tea; tea, bread and butter, and jam form the 4 o'clock meal; and dinner, soup, roast ribs of beef, and some simple pudding, followed by black coffee. This is ample fare, but the want of variety is surprising. Cake at Friday's tea party is indeed a luxury. Two meals are laid for the night sisters in the mess room, consisting invariably of cold meat, pudding, and tea.

### On and Off Duty.

The hours of duty are much the same as at military home stations. Day sisters come on duty at 8.30 a.m.; off duty from 2 to 5 p.m. including tea hour, on duty again till 7 o'clock dinner, a return to the wards then being optional. The night sisters, of whom there are from four to seven as circumstances dictate, come on duty at 10 p.m., and are free at 8 a.m.; but the work is generally over by 6.30. Written or verbal reports are passed on from the day to the night sister. On each of the three landings every afternoon between 2 and 5 p.m. an "orderly sister" takes charge while the rest of the staff are off duty, and that day the specified sister has no off duty time. On the upper landings the turn comes round every third day; on the ground floor every eighth day, as there are many more sisters in charge of big wards there. We have no half days, but the matron of the Irish Hospital superintending here is very kind in giving week ends or two or three days' holiday when the work allows. Any sister at all incapacitated by sickness is at once sent to the "Sick Sisters' Hospital" about two miles away, and, judging from the account of returning invalids, it is a small Arcadia, and offers an inducement "to go sick" in order to get there for a few days.

### The Orderlies.

Captain Mould, R.A.M.C., represents the Army element of the hospital and controls the orderlies. As regards the latter we are much better off than at Netley, as described in your issue of August 25. Each ward of the ground floor has an orderly for day and night duty, and more if required. On the second floor there are 7 to about 150 beds, and on the upper floor a much smaller number to considerably fewer patients. The orderlies, like the sisters, do a week of day and night duty alternately, and though, as some think, not a good plan in itself, as involving a second change just as Nature is adapting herself to the initial one, it is a great improvement on the system obtaining in English stations. Moreover, as the orderlies take duty in one ward continuously, there is this opportunity for them to really know the cases of which they have care. We have only two R.A.M.C. men among the orderlies, who number 102, the others being those of the Irish Staff, drawn chiefly from the Irish Constabulary force—a most capable class of men—regimental men, and a few convalescent patients, who, speaking from experience, sometimes make first-class nurses. The orderlies have Kaffirs to assist in the more thoroughly menial work—cleaning bed-pans and spittoons, sweeping and scrubbing of floors and lavatories. I am told that very few cases of enteric develop among these Kaffirs. All soiled linen is at once taken to a boiler of disinfectant, and all excreta removed from the receiving pail when necessary, and under an orderly's supervision is burnt. Kaffir labour is also employed in the domestic part of the hospital, such as the washing-up of crockery, &c.; and a Kaffir girl undertakes the duty of housemaid.

### The Cases.

The patients are chiefly medical cases—enteric and dysentery—both happily of a milder type than in months past; but there is some anticipation of an epidemic of typhoid at the close of the year, as the spring has been very hot, making it difficult to believe that it was not midsummer. Even the nights have been hot and breathless, and one has been glad of the lightest clothing. We usually wear full indoor uniform when going out for a drive or walk, but dust plays mischief with caps and aprons in very short time. Bonnets and cloaks are quite discarded as unsuitable under the circumstances. We have had our first thunderstorm, but it did not come up to my expectations of a deluge, though the rain was thoroughly tropical—it has made the roads delightful for driving, and cooled the air many degrees. In fact, as I write, on night duty, I am wearing a fur-lined cape with much comfort.

### "To be Turned Out."

Although we came out to hard work, we have been glad of the pleasant recreations that have fallen to our share. Every Friday afternoon the band of the Lincoln Regiment kindly plays for an hour in front of the hospital, and forms an excellent excuse for a tea party given by one of the Irish doctors weekly. Last Friday the tea party was honoured by the presence of General Baden-Powell. He has requisitioned this building for police barracks, and we hear we are to be turned out forthwith, the patients being sent away as rapidly as their illness permits. We are all wondering what will become of us.

### Farewell to the Irish Hospital Staff.

Last week we had a splendid picnic as a farewell to the staff of the Irish Hospital starting for home this week. We drove out in various vehicles, to a farm and just escaped a nasty accident, as a horse bolted, and eventually the Victoria capsized, throwing all its occupants out. No one was damaged enough to turn back, but the carriage lost a wheel. We learned afterwards that while we were picnicing the Boers came over a kopje not four miles distant and captured 300 head of cattle, outposts notwithstanding. By the kindness of the Hon. Rupert Guinness a Cape cart has come twice a day to take the nursing staff for drives in their off-duty time, the night nurses having it from 9.30 to noon, and the day sisters from 2 to 4 p.m. In this way we have seen a great deal of the neighbourhood of Pretoria.

### The Nurse in Hot Climates.

By EDWARD HENDERSON, M.D., F.R.C.S. Edin., late Surgeon, General Hospital, Shanghai.

(*Continued from page* 67.)

*Bowel Disorders.*—The quantity of water used must be regulated according to the special requirements of the case, and the sensations of the patient will usually need to be consulted, though not altogether trusted to, in determining this; under ordinary circumstances two pints can be introduced without causing much inconvenience; if the washing is merely the preliminary to the administration of an opiate, a single pint will generally be enough. In the case of an adult a common prescription for an opiate enema is thirty or forty drops of laudanum in a few ounces of thin, warm starch; but this, of course, must not be given until after all the water used in washing has been got rid of. The small bulk of a starch and opium enema necessitates the use of a small syringe for its introduction. Glass syringes are very often imperfect; the slightest resistance at the point causing the ill-fitting piston to traverse the fluid and leave it behind in the body of the syringe. The nurse should always carefully test the efficiency of a small syringe before using it. Vulcanite syringes are generally well made, but have the disadvantage of not allowing the nurse to see at once when they are not being completely emptied. A small indiarubber ball is, perhaps, the best instrument to use, care being taken fully to express the contents. When nitrate of silver enemata are ordered, and they are frequently prescribed in cases of chronic dysentery, the nurse will be guided in their administration by the instructions she receives from the doctor in charge of the case. Exact details vary somewhat in different hands, but bowel washing is always a preliminary step, done either with plain warm water or with water holding bicarbonate of soda in solution. In giving these enemata the staining properties of the solution should be remembered in connection with bed coverings and sleeping clothes, and provided against by the nurse. Hot fomentations and wet packs are frequently used in the treatment of bowel cases, but it may be safely assumed that the trained nurse has nothing new to learn regarding the value of these or the details of their application, which for all practical purposes are the same in hot climates as in England. If nothing more than warmth and support is wanted, a single or double fold of cotton wadding laid over the abdomen under a broad bandage or cummerbund will do all that is required. For the application of dry heat Manson recommends the use of the flat tin boxes made in Japan, in which charcoal cartridges are burned; the boxes should be encased in flannel.

*Temperature in Bowel Cases.*—I have already spoken of temperature taking as one of the nurse's duties, needing on the whole closer attention in hot than in temperate climates. In many cases of bowel disorder the thermometer gives important indications as to the nature, progress, and possible complications, of the disease. Thus a sudden rise in temperature in acute diarrhœa or dysentry may show the bowel affection to be really due to the poison of malaria; while in a chronic case, a recurring rise of temperature in the afternoon or evening may be for weeks the only symptom pointing to the formation of a centrally placed liver abscess. In cholera the thermometer is only of real service after reaction has set in. In acute dysentery, not connected with malaria or complicated with liver abscess, its indications are not of so much value; with regard to these cases it should be noted that in some of the worst the temperature of the patient may be little above normal throughout the illness. Taken along with other symptoms such as restlessness, delirium, and a failing pulse, a subnormal temperature in dysentery indicates destructive ulceration of the bowel and a fatal termination.

*Feeding in Bowel Cases.*—In all bowel cases the feeding of the patient is one of the most important of the details involved in treatment, and this is a matter in which the nurse is largely concerned. In the acute stage of diarrhœa and dysentery milk is often the only food allowed, while in chronic cases a diet consisting of milk alone is usually given a full trial before recourse is had to other forms of nourishment. In using milk the purity of the supply is the first point which should receive attention. Wherever natives are concerned in the collection and distribution of milk, and the cases are few in hot climates in which they are not more or less employed in this way, purity is endangered, and, speaking generally of dairies throughout the East, can seldom be absolutely depended on. Milk should as a rule be boiled before it is given to the patients. After boiling the milk should be poured into bottles and stored in the ice chest; it is unnecessary to add that the bottles used for the purpose must be kept scrupulously clean. Buffalo milk is seldom used by Europeans; it is richer than cow's milk, and has a less agreeable flavour. Milk is usually taken readily by the patient, but in a few cases it is objected to from the first, and in some, even when taken easily, it is not digested. When milk is not digested curds appear in the motions, and the presence of these should always be looked for in cases where milk is given largely. The following is nearly a complete list of the various plans proposed to improve the digestibility of milk and secure the assimilation of a sufficient quantity:—Dilution with plain water, rice and barley water, effervescing and alkaline waters; the addition of alkalies, and, or, common salt; aeration; concentration by evaporation; predigestion by peptonising. The aeration of milk is done by means of a machine called a seltzogene, but at the present date the same effect may be produced, and possibly more conveniently, by the use of "sparklets." In condensation by evaporation, recommended by Thin in special cases, the milk is put in an enamelled saucepan which fits into another pan containing water, and the heating is conveniently done over a spirit lamp; the milk is never allowed to boil, and is constantly stirred to prevent a skin forming on the surface; a reduction to half the bulk may be ordered, and this will take between one and two hours to accomplish. When milk is the sole nourishment given quantity is a matter of considerable importance; this is, however, of course, a matter to be regulated by the doctor: it is enough for the nurse to know that from three to six imperial pints of undiluted cow's milk in twenty-four hours, in the case of an adult, covers the ground between what is commonly regarded as a small and a large quantity. Milk is usually given in amounts of from five to ten ounces at one time. With a view to improve its digestibility by ensuring slow consumption it has been recommended to give it in some cases sucked through a glass tube or from an infant's feeding bottle. The addition of a little coffee or lightly-infused tea to the first cup of (warm) milk taken in the morning is often highly appreciated by the patient; and this, along with a slice of well-toasted or torrified bread, gives at least the semblance of a breakfast, and breaks the monotony of the restricted diet. Besides milk the only other foods which call for special notice here in connection with bowel cases are beef juice and beef mince. Either of these is frequently ordered, and it may be, as in the case of milk, to the exclusion of any other form of nourishment.

(*To be continued.*)

### A Visit to the Schools at Darenth.

By a Correspondent.

Anyone spending an hour or so in the schools of the Metropolitan Asylums Board at Darenth, will be convinced that at least two things are absolutely necessary before it is possible to become a successful teacher of defective children, viz., enthusiasm and experience. Enthusiasm, because, though the work is not without most encouraging results, it is apt to be disheartening; and experience, because, though the children are taught in classes, each one needs to be known individually before his or her limited powers can be drawn out. It is by no means to be lightly taken up; it is on the contrary the work of a lifetime; and though a teacher may be thoroughly trained, and quite capable of instructing ordinary children, she must unlearn much when she takes upon herself the education of those who are technically called "defectives."

### The Teaching.

The very term "children" has a significance of its own; the young men of twenty-five who are making baskets to order, as neatly, though not as quickly, as an average workman, are still "children" from the medical point of view; and the pupils are classed necessarily according to capacity and not age. The schools are mixed; boys and girls working together, and the plan is found to answer well. "Do they quarrel?" I inquired, and received the reply "Less than ordinary children."

The teaching is based entirely on the kindergarten system —working *from* results, not to them. The first principle is that the child is making something, and so long as he knows this he will work. An experiment has recently been made in teaching the children stitching; after three weeks' trial it was found to be a dead failure, it did not excite the slightest interest. This is to be overcome by making the obnoxious stitch an integral part of a doll's garment, when it will acquire a value in the eyes of the children. The sewing on of buttons was absorbing the attention of a row of boys in the large schoolroom; they had buttons on their coats, and so could see a certain amount of meaning in making detached buttons stay on detached bits of cloth; when these bits of cloth become the trousers of a boy doll, there will be more meaning still in the process.

Macramé work is a good example of the principle rows of children, boys and girls, were deeply absorbed in making edgings of intricate patterns. I spoke to one little girl, and though she answered, she did not look up from her work. But it is only the advanced pupils who can be induced to do the various stitches merely as specimens; and to see something growing under your hand is after all a very human joy.

### How the Children Teach Themselves.

In order to be able to produce something, one little girl had taught herself to knit, out of school, on two broken bone knitting pins which she had found, and both the pins and the knitting—a poor little strip half an inch wide, of crude-coloured wools—were hidden in the bib of her pinafore, and only coaxed out by one of the teachers for the visitor to see and admire; and many children are so keen to learn that they teach themselves to knit on bristles from bass brooms. It was interesting to learn a little of the children's likes and dislikes in colour and form. As one would suppose, reds and pinks have the first place in their affections, then blues and greens, and the duller browns seem to be accepted as contrasts. The letter A was held up for admiration by one girl of perhaps ten years old, drawn in green pencil, and carefully filled in with brown; although she murmured "green" in answer to the repeated inquiry of her teacher, she was too shy or too ignorant —perhaps both—to name the other colour.]

### Some of the Difficulties.

When the children come to Darenth from the parochial infirmaries they are for some time too distrustful of their new surroundings to speak, and it is frequently months before they answer when spoken to even in the gentlest manner. It is thought that this is due to distrust of the teachers, or possibly there may have been long repression or neglect at home; one child who was brought to the schools, and said to be speechless, suddenly began to talk after about two years, and from that time was quite comprehensible.

It has also been noticed that there is no grief, or very little, at parting from home and relations, although deficient children both cry and laugh more readily than normal ones. This may be because the worst of the parting is already over, the children having been for some months probably in the Infirmary before being drafted to Darenth; but, whatever the cause, visiting days are by no means the horror to teachers and nurses that they are in a Children's Hospital. At the same time, they have great capacity for affection; and latterly a nurse from the London Hospital, who had been, eighteen months before, an attendant at Darenth, was recognised and welcomed by many of the children when she paid a visit to the Asylum. Many old pupils who have gone out into the world as soldiers, sailors, and even settlers in the colonies, still write to the Darenth teachers; and even those who are unfit for anything but to be drafted into the adult block, are always pleased to meet the teachers who were kind to them in the schools. Two young men who had been pupils lately bicycled from Stratford to see their old friends.

### The Children's Work.

The basket-making industry has already been mentioned; this department is taught by one of the men who occupy in a sense the position of "fathers" to a certain number of children; that is to say, a few married couples have charge of some of the wards, to give more of a home feeling to the little ones whose own mothers and fathers are far away or perhaps dead; and this man and his wife have the care of a ward. Practical basket-making has only been introduced during the past eighteen months, though ornamental work has long been a staple occupation, and already plenty of orders come in, and a goodly number of well-made baskets go every week to other institutions, or to private purchasers. It takes a skilled workman about an hour to make one of the "prickles" or orchard baskets, and these boys can accomplish one in just double the time; no doubt speed will come with practice. "Basket-weaving" is graded, from paper-work up to cane-chair seating. Much of the work is astonishingly good. Some dainty little models of babies' clothes are beautifully made, the work, with the exception of the cutting out and this it is hoped soon to teach, being done throughout by the girls. One girl of eighteen had made and honey-combed very prettily a blue smocked frock which was quite worthy of exhibition, and it seems a pity that these specimens cannot be shown at some educational exhibition, so that the public may be afforded a tangible proof of what patient, loving teaching may do with these deficient little ones.

There is another branch of industry which may develop in time, viz., hand-loom weaving. The perfectly woven doll's towels, with a red stripe as in "real life," woven on no better frame than the rim of an old slate, show a neatness and dexterity that should have every encouragement; indeed, the experiment of a hand loom would be well worth a trial.

Some of the drawings are excellent, both in pencil and wash; they are neat at the edges, and the free-hand watercolour drawings of birds and wall-paper designs showed great care in copying. There were one or two original designs also.

It is interesting to know that some of the children are already young horticulturists; for the boxes outside the schoolroom windows are tended entirely by them.

THE CHILDREN'S FUTURE.

One thought occurs to the visitor to these most interesting schools, and that is, what becomes of all the knowledge thus laboriously gained when the child leaves school? In some cases, as it has been shown, sufficient intelligence is developed to enable the man or woman to become self-supporting, or partially so, but what of the rest? One is rather led to the conclusion that they lapse into mere children of Gibeon—hewers of wood and drawers of water—scrubbers of floors or low-grade laundry workers.

And one comes away from this isolated colony of "impotent folk" with the feeling that here are many possibilities for future development; the talents entrusted to these little ones are neither great nor brilliant, but a brief visit proves that they are being helped to develop along the line of least resistance; their movements may be slow, but there is great capacity for taking pains.

### Appointments.

NORWOOD COTTAGE HOSPITAL.—Miss E. M. Roberts has been appointed matron. She was trained at St. Thomas's Hospital, and has since been lady superintendent at Monsall Hospital, Manchester.

ROYAL PORTSMOUTH HOSPITAL.—Miss E. Richardson has been appointed assistant matron. She was trained at St. Bartholomew's Hospital. She has since been sister in charge of a home hospital at Eastbourne, sister at the Royal Hospital, Southampton, and has done six months' nursing in South Africa on A.N.R. service.

### Minor Appointments.

DERBY BOROUGH ASYLUM.—Miss Kate Robertson Sinclair has been appointed head nurse and housekeeper. She was trained in the same institution and has recently been housekeeper in Berrywood Asylum, Northampton.

ISOLATION HOSPITAL, WIMBLEDON.—Miss Maude Bateson and Miss Margaret Priestman have been appointed ward sisters. Miss Bateson was trained at Mill Road Infirmary, Liverpool, where she has since held the position of ward sister for three years. Miss Priestman was also trained at Mill Road Infirmary, Liverpool, where she has since been ward sister for three and a half years.

ROYAL HALIFAX INFIRMARY.—Miss Margaret Richardson has been appointed sister. She was trained at the Westminster Hospital for five years and for one year at the Southampton Fever Hospital.

FULHAM INFIRMARY.—Miss Alice Mary Mason has been appointed night superintendent for the female side. She was trained at the Poplar and Stepney Sick Asylum and has since been ward sister in the same institution. Miss Maggie M. Beales, who was appointed night superintendent for the male side of the infirmary last month, was trained at the East London Hospital for Children, Shadwell, and at Guy's Hospital.

MILITARY HOSPITAL, ATHENS.—Miss Grace Button has been appointed sister. She was trained at the Stoke-on-Trent Infirmary, where she also held the post of sister, and she had previously trained in fever nursing at the North-Eastern Hospital, Tottenham.

### Where to Go.

QUEEN'S HALL, LANGHAM PLACE.—Thursday afternoon, November 29th, at 3 (General Sir Redvers Buller in the chair).—Illustrated lecture by Mr. W. T. Maud, special artist of the *Graphic*, entitled, "Four Months in Beleaguered Ladysmith," in aid of the Central London Throat and Ear Hospital, Gray's Inn Road.

### Nursing the C.I.V.'s.

BY A CORRESPONDENT.

READERS of the MIRROR who witnessed the C.I.V.'s procession last week, will be interested to learn that the "Nurse who sat on the box of one of the invalids' carriages" was Sister Ethel Pope, who had been nursing the sick on the voyage. This is the third time she has come home in charge of the sick and wounded from South Africa.

A RED-LETTER DAY.

"It was the proudest day of my life," she said afterwards. "I shall never forget it as long as I live; to think that I should be the fortunate nurse on whom such an honour fell! I looked out for nurses all along, and their smiles were good to see; the cheers of the crowd—'Bravo, Sister!' 'Well done, Nurse!'—were almost too much for me, and brought the tears to my eyes many times as we went through the City. It was a long and tiring day, but I wouldn't have missed it on any account."

"Sister Pugh was not with you?" I asked.

"No; she was not well, and was anxious to join her friends as soon as possible. We had had a rough voyage, and we had both been working hard."

ON BOARD THE "AURANIA."

"Although all the men were convalescent when we left Cape Town, about eighteen of them developed very serious typhoid, mixed with malaria; there was not a typical typhoid chart among them, and we were busy all day and half the night giving ice-packs and frequent spongings to get the temperature down. The ordinary fever temperature was 104 to 105, and in the worst cases it was 107·4, and one was 108·4; these last died; we lost five altogether. It was heart-rending to lose them, and I saw tears in the officers' eyes when they died; there is such a genuine friendly spirit in the corps, they are like a large family, and the men are devoted to the Colonel and other officers."

"How many doctors were on board?"

"The P. M. O. was Major Sleman. I had his patients consisting of mounted infantry and officers; then we had Captains Ryan and Thorn also. All the doctors worked splendidly. We had also a corporal of the R.A.M.C., and the C.I.V.'s own orderlies. Please say a good word for them for they really worked magnificently, and their willingness quite made up for any lack of special knowledge."

"Did you keep to regular hours of duty?"

"No, it was quite impossible; there was so much to do, and all this illness was so entirely unexpected. We had broken nights, too, for you never ceased to feel responsible for your patients when they are so ill as that, and neither of us could be spared for night-duty. Of course we never left them alone for a moment; and at night sentries were told off to watch delirious patients—a sentry, or sometimes two, to each case—and I used often to slip on a dressing-gown and go down during the night."

"What do you think of the C.I.V.'s as patients?"

THE VOLUNTEERS.

"They are so plucky and brave; even with the worst symptoms of enteric, they would pretend they were quite well. They have borne all the hardships of the campaign magnificently, and it is thought that it was the reaction that brought on all this illness; they were not accustomed to rest and quiet, and they collapsed. The C.I.V.'s had a fine time at St. Vincent; the King of Portugal had sent a message saying that they were mostly dukes and millionaires, and were to be treated as such; and this was a tremendous joke with them! They were really royally treated. But I didn't land; it was at St. Vincent that one of our men died. One had died just before reaching there, and after leaving we lost another. Then a fourth died on the Thursday before we reached Southampton, and the fifth, saddest of all, early on Monday morning as we lay in Southampton Water. Besides the lying down cases—the worst of whom were sent to Netley, a good many men being treated as out-patients—I had over twenty at one time; I saw them twice a day, when they came to the dispensary for medicines. In the 'wards' there were forty beds, the berths being five-cabined ones. Bunks are very awkward, as of course you can't get to the other side; but things were made as convenient as possible, and if a man who was seriously ill was in an upper berth, we moved him down."

### Echoes from the Outside World.

AN OPEN LETTER TO A HOSPITAL NURSE.

Very little is known in England about the Queen of Portugal, but evidently she is both strong and courageous. The Portuguese Royal Family have been staying at the seaside resort of Cascaes, and, although we have now entered the month of November, the Queen had been bathing regularly each day as usual. She had resumed her walking dress when she noticed that her boatman, who had put out to sea again, had met with an accident, and was in the water with his boat on the top of him. Without losing a moment or waiting to divest herself of her outer garments, she swam to his rescue and brought him safely to shore. It would probably have gone hardly with the man had not the Queen been in the vicinity, for his leg having been broken he had no chance of helping himself. He was carried to the Palace, and remains under the treatment of the Court Surgeon.

Dear old "Bobs"! We had imagined that our admiration had been gradually growing until it had attained its height and could go no further, when suddenly his circular letter to the English nation, dated Pretoria, September 30, 1900, is published, and we find that our enthusiasm is redoubled at once. Truly he is great man in more than one sense, and no wonder the army idolises a chief who can write of his soldiers as Lord Roberts does in his appeal to his country men and women at home. It is not only the words of the letter which impress, but reading between the lines one can see the Christian fellowship, the kindly thoughts, and the real desire for the good of his men which has animated the commander, who now nearly a year ago roused himself from the natural grief he felt for the loss of an only son, and came to his country's aid in such a strikingly successful manner. In begging the public to refrain from "treating" the men in public-houses or in the streets upon their return, he says: "From the very kindness of their hearts, their innate politeness, and their gratitude for the welcome accorded them, it will be difficult for the men to refuse what is offered to them by their too generous friends." Then having proudly stated that nothing deserving of the name of *crime* has been done in South Africa by the army, he continues: "There has been no necessity for appeals or orders to the men to behave properly. I have trusted implicitly to their own soldierly feeling and good sense, and I have not trusted in vain. They bore themselves like heroes on the battlefield and like gentlemen on all other occasions." This last sentence which is sure to become historic, might well be placed before each recruit as the standard to which he should strive to attain.

Politics are not in our line, are they? But the numerous changes which have been made in the Government are not without interest to most women. Of all the many statements I have seen or heard, I think that which has impressed me most is the story of how, according to gossip, the Duchess of Devonshire has only just missed having the desire of her heart gratified. It is said that Lord Salisbury went to Balmoral prepared to relinquish the Premiership to the Duke of Devonshire, under whom he was willing to serve as Foreign Secretary, but that the Queen wished him to continue at the head of affairs. Of course, the Queen's wish was law, and one does not wonder that Her Majesty is anxious that a statesman to whom she has been so long accustomed should remain Premier. But all the same, one can sympathise with the Duchess, who would certainly, as the wife of First Minister of the Crown, entertain her guests with becoming splendour at the noble mansion in Piccadilly. It is amusing to note that all the complaints against Lord Lansdowne have only ended in his promotion to the great post of Foreign Secretary, because, as one paper puts it, he can speak French well. We have also got a new Home Secretary in the person of Mr. Ritchie, who is not a lawyer, but a capital man of business; while Lord Selborne, the Prime Minister's son-in-law, and the son of an equally distinguished man, is First Lord of the Admiralty, and Mr. St. John Brodrick takes Lord Lansdowne's place at the War Office. Handsome Mr. Wyndham is to be Irish Secretary, and it seems probable that he will have a busy time with the Nationalists in the House of Commons.

The subject of intemperance, and any measures which can be taken to reduce the evil consequences of the habit, must be of especial interest to nurses, because in the course of their work they of necessity see so much illness which has been brought about by over-indulgence in alcohol. The inebriate homes, of which there are very few at present, have hitherto been of limited good, because, owing to their being always full, it has only been possible to gain admittance after long waiting, and then almost as a favour. The London County Council opened one home and expressed their willingness to do more; but magistrates, finding it impossible to send suitable cases to a home from lack of sufficient accommodation, have reluctantly been obliged to fine or imprison the delinquents, allowing them afterwards to return to their evil ways. But now the Home Office has apparently decided to take action in the matter, and both stipendiaries and the great unpaid have been requested to furnish, through their clerks, full particulars of all cases brought before them of eligible dipsomaniacs for inebriates' homes. Two women who came within the category of habitual drunkards were remanded at Southwark Police Court one day last week in order that they might be committed to a home. They protested loudly, but Mr. Slade was inexorable, and intimated that in future prisoners would have no choice in the matter, and a great blessing too! Perhaps after this compulsory retirement has been in force for some time the stigma which at present rests upon our sex—that there are two women drunkards to every man—may be removed.

I do not think that bodily cruelty is often practised by a woman on her young servant, because most women are so anxious to keep their domestics that they do all they can to conciliate them; but that such people as servant-beating mistresses do exist was proved by the notorious case of Mrs. Nicholls, who starved and beat Jane Popejoy to death some time back; and now a Mrs. Cranstone, the young wife of an elderly builder, has followed in her footsteps. The girl was an orphan of seventeen, and she had been some nine months in the employ of the "lady," who slapped her face about three times a week, beating her with a stick, a poker, and a hairbrush till at last the master of the house himself sent the servant away to his son's residence. The defence that the injuries were not caused by calculated cruelty, but were the result of a hasty temper, did not save the prisoner from being sentenced to three months' hard labour. It is to be hoped that during those three months Mrs. Cranstone will learn how to control the temper which has led her into such trouble.

### A Book and its Story.

A ROMANCE OF REAL LIFE.[1]

THE authoress of "Foreign Courts and Foreign Homes" has in her present book of personal and historical memories brought together some very interesting reminiscences of a past century, and also of those extending far on into the present. Her early life was spent abroad, and it was from eye-witnesses of the scenes described that she received them. The personal incidents are connected with interesting people, whose life history is in many instances tragic, and all instances pathetic, with the pathos inseparable to victims of cupidity or tyranny on the one hand, or when sacrificed to the call of honour or duty on the other, in devotion to those who were, in many cases, unworthy of the sacrifice.

Among the most interesting are the anecdotes told to the authoress by her father, whose memory carried him back to the close of the First Empire. His friendship with the Duc de Morny brought him into touch with Imperial history, and from the Duc he learned many details of Josephine's life, and that of the beautiful Queen Hortense, her daughter, married to Buonaparte's brother, Louis Buonaparte. These reminiscences are from stories told to him by a devoted old family servant. The Duc was half-brother to Napoleon III. He was a man of many gifts, and physically and intellectually his brother's superior. He held important posts in his Government, and, had he survived, "it is considered he would never have allowed the Emperor to embark on the fatal war with Germany." The brothers shared one common feeling of devotion to their lovely mother —a devotion amounting to veneration and idolatry, peculiar to Frenchmen. She had, as is well known, apart from mere physical beauty, the fatal faculty of fascination, and like so many women thus gifted, her life was one of singular unhappiness. An intuitive perception of coming events, amounting almost to prophecy, was also one of her characteristics. To Josephine she clung with all the tenderness of a heart, passionate and proud, wounded in its own quest for love by her union with a brutal, unsympathetic consort. Perhaps her experience of the sorrows associated with an ill-assorted marriage and the sufferings incidental to it, may have quickened her prophetic perceptions on behalf of her beloved mother. For strung to a pitch bordering on frenzy by Napoleon's treatment of her, and the announcement of the ntended divorce, against which her prayers and entreaties were futile, she exclaimed, at a last interview with him, "You may divorce my mother, separate from her; but to the last hour of your life no woman will ever surpass her in your affections." "Strange to say, her prophecy," added the Duc de Morny, "came true, for on Napoleon's death-bed her image was ever before him; and though historians say his last words were 'Tête d'armée,' I was told by an eye-witness that his last faint whisper was 'Joséphine.'"

There are painfully vivid recollections of the Revolution. Many of those connected with Marie Antoinette and the Royal family will be new to readers. They gain an almost weird interest from being related by an elderly lady, known to the authoress by her convent name of "La dame Blanche," or Sister Louise. The nationality of the gentle lady of sorrow, who became prematurely old from the terrible sights and sufferings she had undergone, was unknown to the sisters of the convent. She was given into the special keeping of the authoress's sister during a summer spent with the nuns in their quiet cloister, situated in an old French town. "What a life hers had been! Her mother had come over to France with the young archduchess, Marie Antoinette's sister, and Sister Louise must have been about the same age as Marie Antoinette's eldest son. She was the constant companion of the royal children, and did many lessons with them—German, especially, which she spoke fluently, naturally."

[1] "On the Banks of the Seine." By A. M. H. Longmans. 6s.

There are pictures of the unclouded days of childhood of the Royal children, whose greatest delight was to go up and down the Seine in the gilded barge which was built expressly for them. Much of her time was spent with the little Dauphin, called by the courtiers, Saint Louis, from his resemblance to his illustrous ancestor. "La dame Blanche" assisted in the flight of the Royal family to Belgium.

"It was the last time I was ever to see the Queen," she added, bursting into tears. "It was quite dark when my mother and I were taken to the little Prince's room by a trusty maid. Obedient to a whisper from the Queen I took the frightened boy on my lap, telling him to put on his *cousine's* clothes; boy like, he felt the disgrace, but as they whispered it was to help his mother he felt that he must obey. 'Saint Louis is looking down—is praying for you,' I said.'"

In darkness and silence the fugitives left the palace, La dame Blanche, at a command from the Queen, assuming the Dauphin's clothes—as it was considered safer for her to pass out in this disguise.

"'Adieu,' said the Queen, when all was ready. '*Priez pour nous.* Then we knelt and kissed the outstretched hand. Then I turned to say good-bye to my Royal playmate, *mon petit* Saint Louis, saying . . . 'Adieu'; he clung about the neck of his *cousine*, whispering tender, childish words, known only to us two. The door opened, and I followed my mother out into the gallery, and never again in this world did I see *mon petit* Saint Louis, for whom, girl as I was, I would willingly have died."

Of the sufferings and terror of the days that followed there are realistic pictures. Too terrible, with all the force of simple truth, to dwell upon, and were it not for the noble deeds of heroism which shine out of the lurid darkness, with bright gleams of undying glory, one could hardly bear to read them.

One last reminiscence of La dame Blanche has especial interest for English readers. It has reference to Henry of York, the last of the Stuarts—a Cardinal of the Roman Church. "My whole heart went out to the Cardinal; he was so handsome and saintly looking. . . . He asked, to my surprise, if I knew that he himself was the rightful King of England, Henry IX. I did not know this, and replied, 'Monseigneur, you look like a king.' I saw him once again, and that was at a grand procession held at St. Peter's at Rome, when the Pope entered surrounded by his Cardinals. First and foremost, with a far-away look in his eyes, was Henry of York. People said he had no ambition to be King of England, preferring to be Cardinal of Rome, where he was much loved and respected. Of all the Stuarts he was the most devoted adherent to the Church. Henry of York is so little known to most English people that perhaps these trifling incidents (of which we have only given an outline) may be of interest to many who still love and revere the memory of that race of kings."

"On the Banks of the Seine" would be an admirable gift book for young people, and it will have special interest for readers who prefer the romance of real life to the unreal fiction which too often masquerades as its representative.

### To Nurses.

WE invite contributions from any of our readers, and shall be glad to pay for "Notes on News from the Nursing World," or for articles describing nursing experiences, or dealing with any nursing question from an original point of view. The minimum payment for contributions is 5s., but we welcome interesting contributions of a column, or a page, in length. It may be added that notices of entertainments, presentations, and deaths are not paid for, but, of course, we are always glad to receive them. All rejected manuscripts are returned in due course, and all payments for manuscripts used are made as early as possible at the beginning of each quarter.

### Everybody's Opinion.

[Correspondence on all subjects is invited, but we cannot in any way be responsible for the opinions expressed by our correspondents. No communication can be entertained if the name and address of the correspondent is not given, as a guarantee of good faith but not necessarily for publication, or unless one side of the paper only is written on.]

### LECTURES BY "PRO'S."

"ANON" writes: I too have been interested in reading about the "Pro's" suggestion and the reply it called forth. May I speak from my own experience? In my "Pro" days—which I spent in a small hospital—I enjoyed the lectures much because each of the medical men who lectured devoted some ten to 15 minutes at the end of each to those who wished explanations, and any "Pro" was at liberty to ask any question, so all benefited. In a larger hospital, which I went to later, the "Pro's" had some real good help from the sisters who took classes on the subject of the weekly lecture and so helped us out of our difficulties.

### THE SPARTAN NURSE IN PRIVATE WORK.

"ONE who looks on also," writes: In reply to "One who looks on," press of work has prevented me writing on this subject till now. I can only say that at the same time I was looking on also, at a case so closely resembling the one mentioned, that I cannot help wondering whether it is not the same. If so, it seems only just to put the other side of the case. This patient, too, complained of backache. The nurse *did* put cushions, &c., and made her most comfortable, and this not once during the night, but perhaps twenty times, about every five or ten minutes, and then said, "try to wait a little, and try to get a little sleep." As to washing the patient's face, it was the nurse who *did* know that it would help to induce sleep and refresh the patient, and who had at first coaxed her to submit to it, though she did not wish it done. Then afterwards, the moment she had eaten her supper, the patient said, "now I will be washed." But the nurse, knowing that it would be better for the quickly-devoured dinner to digest, and that it was then only about 7 P.M., suggested the patient should rest a little first. This was the other cause for anger.

### VISITING NURSING FOR THE MIDDLE CLASSES.

"B." writes: With reference to the articles which have lately appeared in the MIRROR on visiting nursing, it seems to me that those who exhibit such kindly feeling for educated working women do not sufficiently realise that the visiting nurse is also a working woman, and that she finds it just as difficult to earn guineas and meet her weekly expenses as other working women. The work of a visiting nurse is most uncertain; her services are not required every day—in fact, a week or two often passes without her having any work at all. The question of fees is quite as much of "vital importance" to her as it is to the patient, in fact, more so, as the nurse may take the patient's illness and so be prevented for a time from earning any fees at all. The private nurse has all her expenses paid while she is at a case, but the visiting nurse has to pay her expenses out of what she earns. By showing approximately what these expenses are, it will prove that a visiting nurse will not be likely to make a very large fortune, however hard she may work:—Board and lodging, per week, 17s. 6d.; rent of box in box-room, 3d.; rent of hanging cupboard, 4½d.; baths and boot-cleaning, 6d.; glass of milk and scone mid-morning, 1s. 2d.; laundry, 2s. 6d.; uniform and necessary clothing, *at least* £12 a year, 5s.; total expenses for week, £1 7s. 3½d. I should think a far better "solution of the difficulty" would be to start a sick fund to which educated working women could pay so much a year, and out of which the nurses' fees could be paid. In this way charity would neither have to be given or received. In conclusion, I would add that it is always best to consider both sides of a question and to bear in mind that "every creature exists for its own sake. Cork trees do not grow merely that they may stop our bottles."

### Uniform on Board Ship.

By AN ARMY NURSE.

### ONE VIEW OF THE QUESTION.

THE vexed question of "Nursing Uniform" seems still to invite discussion; another and quite recent aspect of the subject may therefore be worth a little consideration. Why nurses should wear uniform on board ship is surely a problem which every sister bound for Cape Town has asked herself, and the solution—if any has been found outside regulation orders—could hardly have been one which would stand the strong light of common-sense upon it.

#### THE RANK OF NURSING SISTERS.

The silly argument used by some to defend the wearing of uniform on board ship, "that as Tommy Atkins, who serves his Queen and country, always wears *his* uniform, nurses when in Government employment always should do so too," quite ignores the facts of the case—that nursing sisters rank as subaltern officers. Lieutenants in company with their official superiors are not required when off duty—custom has virtually made this law—to appear in uniform, except when under arrest. Indeed, to follow this argument to its logical conclusion, it would be reasonable to assume that sisters on their way to South Africa were in this unhappy condition.

Before going further into details, it should be admitted that orders are orders and require obedience; but while recognising this, the belief grows upon one that those who framed the rules overlooked the fact that they would operate in quite other circumstances than those in which they were conceived.

#### THE OBJECTIONS.

Conceding that it is well for nurses to wear uniform when off duty in England—still an open question among authorities—it is quite another matter to apply that rule to travellers on board ship, in a tropical climate, under circumstances when a renewal of clean uniform is out of the question, when the exigencies of the case demand that at the end of the journey the sister should be prepared to go at a moment's notice anywhere, without reflection on the state of her kit. Moreover, in addition to all these serious and real objections, the sister frequently receives no official instructions that she is required to appear in uniform till she arrives on board, with uniform packed in boxes labelled "Not wanted on voyage," and safely deposited in the hold of the ship, and plenty of mufti in her cabin boxes, anticipating a pleasant holiday away from everything suggestive of sickrooms and patients. For it must not be forgotten that to many of the A.N.S.R. "calling up for duty" by the War Office has meant the relinquishment of the yearly vacation, and the sea voyage to the Cape has had, perforce, to be the only break in perhaps eighteen months or two years of hard work.

#### ONE POSSIBLE PLEA.

There has been much discussion as to the appropriateness of nursing uniform at places of amusement. The defenders of such a proceeding would surely find it difficult to produce any argument for its suitability on board a mail steamer. One possible plea suggests itself; but the nurse who does not respect *herself* is not likely to be influenced by the consideration of bringing adverse criticism or disgrace on her uniform—indeed, in justice to all self-respecting nurses, and in view of the many unworthy counterfeits, it would be well if outdoor uniform were entirely discarded by the nursing profession. Various suggestions have been made as to regulation and registration as a means of solving the problem, but the hard-and-fast definition of a trained nurse of three years' certificate would probably be unworkable, and certainly unjust. Very little would be lost if outdoor uniform were abandoned, and the inconvenience of changing into mufti would be far more than counterbalanced by the absence of conspicuousness, the real sense of being off duty, and the knowledge that other people's bad behaviour could not bring odium on a class of women who, in the main, are of good character and self-respecting.

#### A SUGGESTION TO THE AUTHORITIES.

To return for one moment to this new aspect of the uniform question, it is to be hoped that the authorities will see their way to making the wearing of it optional on board ship on the return voyage from South Africa.

### For Reading to the Sick.

BY love serve one another.—*Gal.* v. 13.

"We can only elevate ourselves towards God through souls of our fellow-men."—*Mazzini.*

How was He,
The Blessed One, made perfect? Why, by grief—
The fellowship of voluntary grief—
He read the tear-stained book of poor men's souls
As I must learn to read it.—*C. Kingsley.*

Brother, we are surely bound
On the same journey—and our eyes alike
Turn upward and onward, wherefore, now thou risest
Lean on my arm, and let us for a space
Pursue the path together!—*Buchanan.*

Be noble! and the nobleness that lies
In other men, sleeping, but never dead,
Will rise in majesty to meet thine own!
Then wilt thou see it gleam in many eyes,
Then will pure light around thy path be shed
And wilt never more be sad and lone!—*Lowell.*

God so loveth us that He would make all things channels to us and messengers of His love. Do for His sake deeds of love, and He will give thee His love. Still thyself, thy own cares, thy own thoughts for Him, and He will speak to thy heart. Ask for Himself, and He will give thee Himself. Truly, a secret hidden thing is the love of God, known only to them who seek it, and to them also secret, for what man can have of it here is how slight a foretaste of that endless ocean of His love!—*E. B. Pusey.*

Let us be silent as to each other's weakness, helpful, tolerant, nay, tender towards each other. . . . May we put away from us the satire which scourges and the anger which brands: the oil and wine of the good Samaritan are of more avail.—*Amiel.*

We may, it we choose, make the worst of one another. Everyone has his weak points; everyone has his faults: we may make the worst of these; we may fix our attention constantly upon these. But we may also make the best of one another. By loving whatever is lovable in those around us, Love will flow back from them to us, and life will become a pleasure instead of a pain; and earth will become like heaven; and we shall become not unworthy followers of Him Whose name is Love.—*Dean Stanley.*

We shall not love Heaven more for loving earth less; the needful thing is not that we abate, but that we consecrate, the interests and affections of our life. Who can doubt that *that* is the truest duty to God which permits us the most disinterested heart for each other; *that* the purest devotion which sanctifies and not chills our affections?—*Martineau.*

It's everybody's business,
In this old world of ours,
To root up all the weeds he finds,
And make room for the flowers;
So that every little garden,
No matter where it lies,
May look like that which God once made,
Calling it Paradise.

It's everybody's business
To search for Heaven's gate,
And to do it with an earnest mind,
Lest it should be too late.
And if he would a welcome gain
From Angels round the throne,
He'd better take his neighbour's soul,
To stand beside his own.—*W. J. Mathams.*

### Notes and Queries.

**The Editor is always willing to answer in this column, without any fee, all reasonable questions, as soon as possible.**

**But the following rules must be carefully observed:—**

**1. Every communication must be accompanied by the name and address of the writer.**

**2. The question must always bear upon nursing, directly or indirectly.**

**If an answer is required by letter a fee of half-a-crown must be enclosed with the note containing the inquiry.**

*Urine Testing.*

(59) Will you kindly give me the name of an easy book or pamphlet on urine testing, suitable for probationer.—*R. D. M.*

You will find all you require in Dr. J. A. Watson's "Handbook for Nurses."

*Sanitary Inspector.*

(60) Being anxious to qualify as lady sanitary inspector will you kindly tell me where to apply and how to prepare for a certificate?—*M. S. J.*

Apply for full particulars to the Sanitary Institute, 72 Margaret Street, W.C

*Training.*

(61) Would you tell me how to prepare to become a nurse? I am only nineteen, and have had no experience, but I am anxious to begin at once. —*Alice M.*

You cannot do better than study domestic economy, that is housekeeping, cooking, needlework, and book-keeping for the next two years. When you are 21 you can become a probationer in a children's hospital. Matrons are always glad of probationers with good groundwork of domestic training.

*Domestic Servant.*

(62) I am anxious to find a home for a servant who has been in the family for 24 years. She is suffering from fibroid tumour, which at times makes her dependent on the care of others. We are prepared to pay £40 a year for her in a home where she would receive sufficient care and have a certain amount of liberty.—*A. F. B.*

St. Peter's Home and Sisterhood, Mortimer Road, Kilburn, N.W. President the Archbishop of Canterbury. A small number of incurables admitted, payment 8s. to 10s. a week. Woodside Home, Whetstone, N. Hon. Sec., Lady Powell. Payment £7 to £22 a quarter. Staff of nurses kept. Some convalescent institution would probably be glad to take in such a case on payment, especially if she could do some sort of work when not laid up.

(63) Is there more than one hospital at Cheltenham? If so, what are their names, and how many beds have they?—*Phebe.*

Cheltenham Eye, Ear, and Throat Hospital (3 beds). Cheltenham General Hospital and Dispensary (82 beds). Cheltenham Home for Sick Children (26 beds).

*Young Probationer.*

(64) Can you tell me of any hospital willing to train a probationer of 18? I intend taking the L.O.S. when 21, and would like three years' general nursing first. I have written to two or three hospitals mentioned in "How to Become a Nurse," but they will none of them do.—*A. B. C.*

Should not the last phrase be "I will do for none of them"? You cannot get training in general nursing worth anything until you are much older.

*Visiting Fees.*

(65) 1. May I trouble you to tell me the usual charges made by a visiting nurse, per hour, per visit, for operations, for single nights, and for laying out the dead? 2. Has this kind of nursing been carried on with any degree of success, and would it be a good plan to send printed circular of fees, &c., to local doctors?—*Quietness.*

From 2s. 6d. to 3s. 6d. per visit, or per hour; from £1 1s.; from 7s. 6d.; from £1 1s. Fees vary according to locality and circumstances of patients. 2. Yes. 3. Certainly. It is usual to have a neat card, containing all requisite information, to send to local medical men.

*Medical Dictionary.*

(66) Can you recommend a small book of Latin medical terms suitable for a beginner; also a pronouncing medical dictionary?—*A. M. M. L.*

Miss Honnor Morten's little volume, "The Nurse's Dictionary of Medical Terms and Nursing Treatment" (Scientific Press, price 2s.) appears to be just what you want.

*Surgical Homes.*

(67) Can you give me the names of, and tell me which are the chief surgical homes in London; and also say which are considered the best in Harley Street?—*Florence.*

It is obviously impossible for us to draw comparisons between the innumerable private institutions of the kind.

*Enterprise.*

(68) Could you give me an estimate of the capital required to start a private nursing home for paying patients in the West End of London or in a country town? What could one do in that way with £500 and furniture?—*Emma.*

This is a matter on which it is utterly impossible to give advice. All we can do is to repeat a very urgent caution to those who wish to embark in a very risky enterprise.

### Standard Books of Reference.

"The Nursing Profession: How and Where to Train." 2s. net; post free, 2s. 4d.
"The Nurses' Dictionary of Medical Terms." 2s.
"Burdett's Series of Nursing Text-Books." 1s. each.
"A Handbook for Nurses." (Illustrated.) 5s.
"Nursing: Its Theory and Practice." New Edition. 3s. 6d.
"Helps in Sickness and to Health." Fifteenth Thousand. 5s.
"The Physiological Feeding of Infants." 1s.
"The Physiological Nursery Chart." 1s.; post free, 1s. 3d.

All these are published by THE SCIENTIFIC PRESS, Ltd., and may be obtained through any bookseller or direct from the publishers, 28 and 29 Southampton Street, London, W.C.

